# Neonatal duodenal atresia with heterotopic pancreas: a case report and literature review

**DOI:** 10.3389/fped.2025.1655746

**Published:** 2025-11-03

**Authors:** Yinmin Sun, Siqi Li, Shiqi Liu, Yufeng Li, Dongwen Quan

**Affiliations:** ^1^ The Second Clinical Medical School, Shaanxi University of Chinese Medicine, Xianyang, Shaanxi, China; ^2^ Department of Pediatric Surgery, The First Affiliated Hospital of Xi'an Jiaotong University, Xi'an Shaanxi, China; ^3^ Department of Pediatric Surgery, Guilin Maternal and Child Health Hospital, Guangxi, Zhuang Autonomous Region, China; ^4^ Department of Gastrointestinal Surgery, Sichuan Provincial People’s Hospital, University of Electronic Science and Technology of China, Chengdu, Sichuan, China

**Keywords:** congenital duodenal obstruction, heterotopic pancreas, pediatric surgery, neonate, children

## Abstract

**Background:**

Heterotopic pancreas (HP) is a congenital anomaly characterized by pancreatic tissue entirely separate from the orthotopic pancreas, lacking direct ductal communication and vascular continuity. HP is most frequently found in the upper gastrointestinal tract, particularly the stomach, duodenum, and proximal jejunum, while involvement of the mesentery is relatively rare

**Case presentation:**

We report a case of congenital duodenal atresia with concomitant HP in a 3-day-old neonate. Emergency laparotomy was performed at 72 h of life for membranous duodenal atresia; web excision and duodenoduodenostomy were completed. Preoperative evaluation showed no other associated malformations (e.g., congenital heart disease or Down syndrome). Intraoperatively, a 10.0 × 6.0 mm focus of HP tissue was unexpectedly found at the jejunal mesenteric boundary, approximately 5–7 cm distal to the duodenal obstruction, necessitating *en bloc* resection with a segment of adjacent bowel. Histopathology confirmed HP. During 12-month postoperative follow-up, the neonate remained clinically stable, with physical development consistent with age-matched normal children, and no disease recurrence.

**Conclusions:**

Given the potential association between HP and duodenal atresia in asymptomatic cases, we propose systematic screening for HP in all neonates with this condition. If identified, concurrent prophylactic resection of the HP lesion during the repair of duodenal atresia is recommended to mitigate the risk of long-term complications, including pancreatitis and gastrointestinal bleeding.

## 1 Introduction

Congenital duodenal obstruction (CDO), occurring in approximately 1.5 per 10,000 live births, requires early surgical intervention. The co-occurrence of CDO with heterotopic pancreas (HP) represents an exceptionally uncommon clinical presentation. HP refers to pancreatic tissue located outside the native gland, lacking vascular or ductal continuity with the orthotopic pancreas (1). Most individuals with HP remain asymptomatic during life, making its true incidence difficult to determine. Autopsy studies report a prevalence of 0.55% to 15% (1–3), while intraoperative and endoscopic detection rates are approximately 0.18% to 0.2% (4). Due to its frequent subclinical course, HP is often identified incidentally during histologic examination. Reported HP distribution varies across studies due to the absence of systematic evaluation; however, available literature indicates that HP primarily occurs in the stomach, duodenum, jejunum, and Meckel's diverticulum (1, 4). Extraintestinal occurrences (e.g., mesentery, greater omentum, spleen) are significantly rarer, with only isolated cases reported (3, 4).

Herein, we detail a rare case of congenital duodenal atresia associated with jejunal mesenteric heterotopic pancreas (MHP). The neonate required emergency laparotomy 72 h post-birth for CDO. Intraoperatively, HP tissue was incidentally identified at the boundary between the jejunum and its mesentery.

## 2 Case description

A female 35 + 5-week preterm infant was delivered vaginally following premature rupture of membranes. Antenatal ultrasonography revealed gastric and duodenal dilation with the characteristic “double-bubble sign” and polyhydramnios, prompting admission for suspected CDO.

The mother attended regular prenatal visits. At 32 weeks' gestation, ultrasound demonstrated polyhydramnios and a double-bubble sign, suggesting possible CDO. With no other anomalies detected, she continued routine monitoring. At 35 ^+^ ^5^ weeks, premature membrane rupture resulted in vaginal delivery of a 2,300 g female infant. Apgar scores were 9, 10, and 10 at (1/5/10 min). After birth, the newborn was assessed, with unremarkable findings on evaluation of vital signs, mental status, and physical abdominal examination. At 12 h after delivery, a trial feeding under close monitoring was initiated, beginning with clear water followed by breastfeeding. After increasing the volume of breast milk, the infant developed mild milk vomiting approximately 2 h later. The frequency of emesis increased progressively, and the vomitus transitioned to bilious yellow-green fluid without a feculent odor (5–10 ml/non-projectile episode). Given the prenatal ultrasound findings, radiography was promptly performed. The infant did not pass meconium within the first 24 h and had only two urinations (∼5 ml each), which were without hematuria or mucus. Clinical assessment revealed afebrile status, alertness, and no lethargy/irritability.

**Figure 1:**
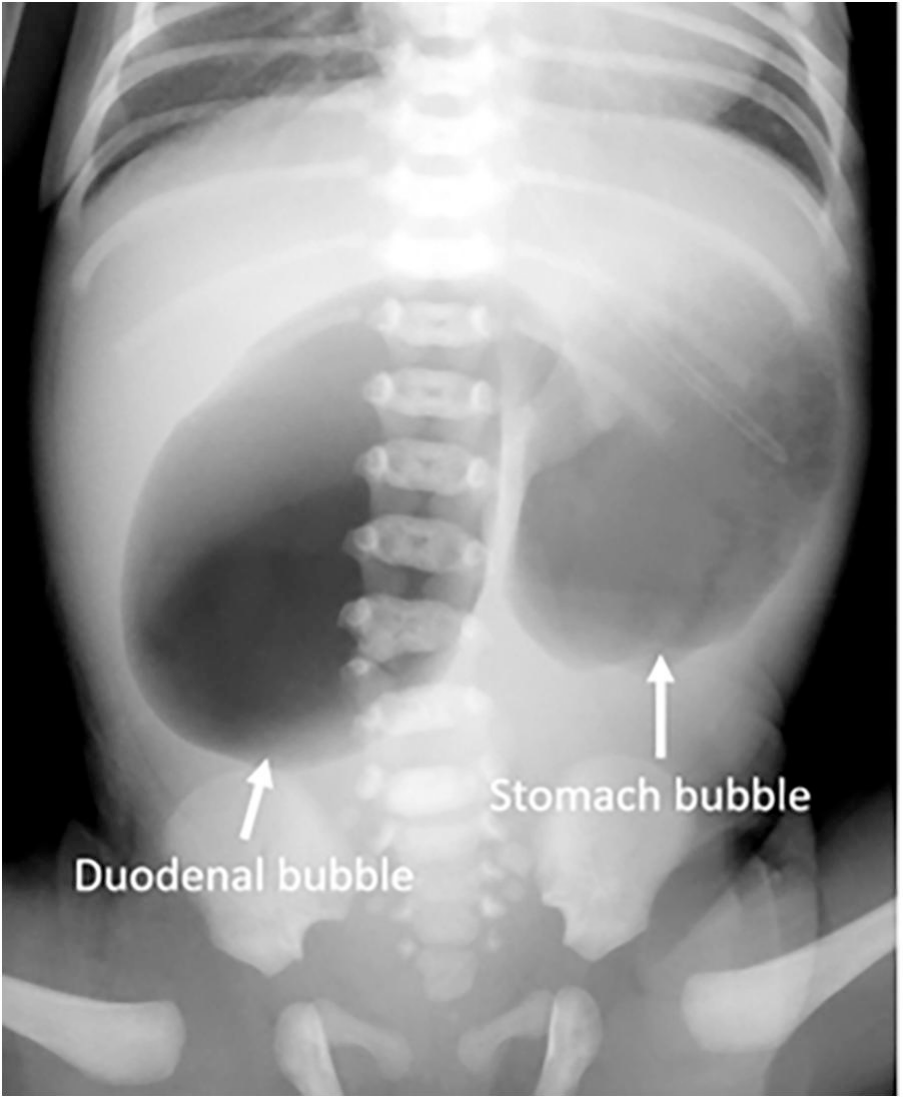
Preoperative x-ray shows the “double bubble” sign with no gas in the distal bowel (arrow).

Admission laboratory investigations demonstrated the following hematologic and biochemical profiles: white blood cell count (WBC) 16.5 × 10⁹/L (55% neutrophils, 30% lymphocytes); red blood cell count (RBC) 5.2 × 10^12^/L; hemoglobin (Hb) 170.0 g/L; and platelets (PLT) 250.0 × 10⁹/L. Serum electrolytes were sodium (Na^+^) 138.0 mmol/L, potassium (K^+^) 4.0 mmol/L, and chloride (Cl^−^) 98.0 mmol/L. Preoperative serum amylase was 78.0 U/L (reference range: 25–125 U/L) and lipase was 55.0 U/L (reference range: 0–190 U/L). Hepatic and renal function panels were within normal limits. Abdominal radiography revealed marked gastric and duodenal dilation with the classic “double-bubble” sign (Figure 1). These radiographic findings, in conjunction with the clinical presentation, were highly suggestive of congenital duodenal atresia. Preoperative examinations revealed no other associated malformations (e.g., congenital heart disease or Down syndrome) in the neonate.

**Figure 2:**
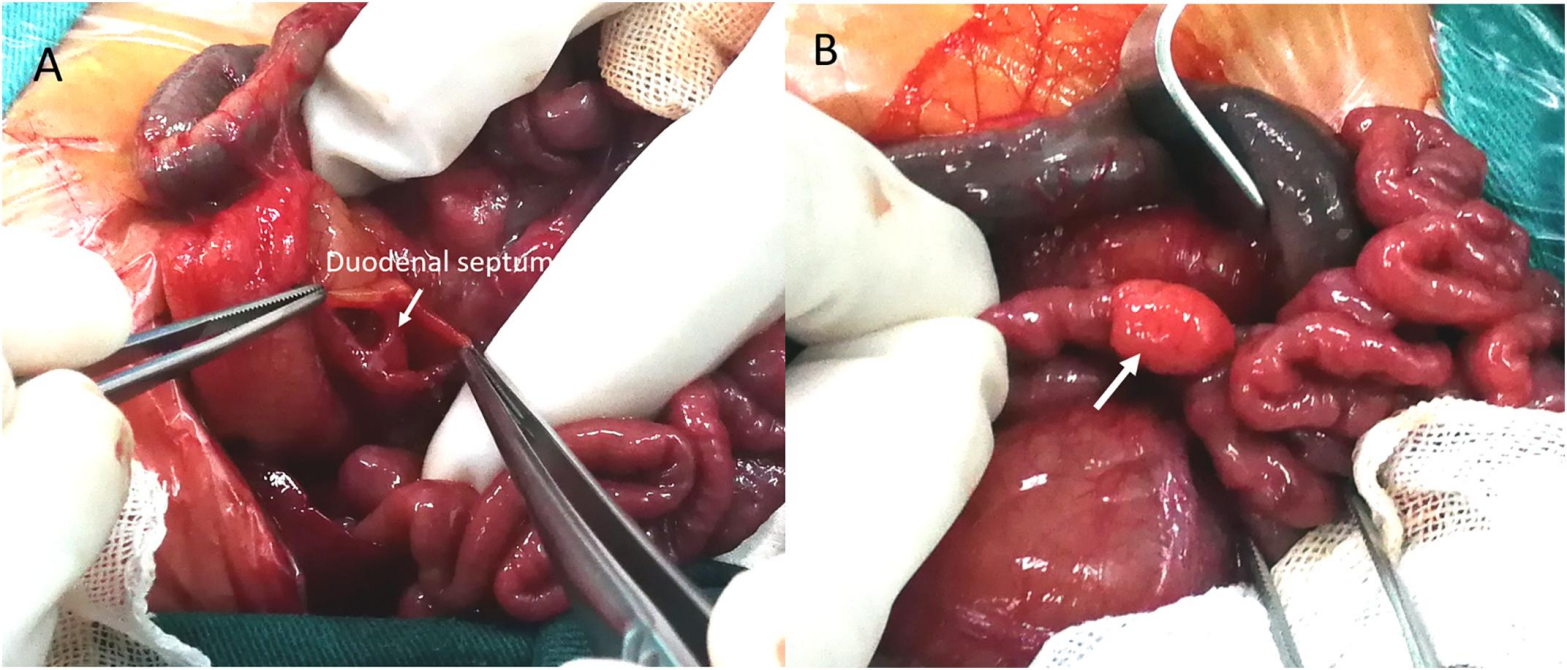
Intraoperative findings. **(A)** Surgery confirmed duodenal obstruction caused by a membrane (arrow). **(B)** A photograph of the HP mass *in situ* shows a 10.0 mm × 6.0 mm yellowish soft tissue mass located at the boundary between the jejunum and mesentery, adhering to the serosal surface of the jejunum (arrow).

**Figure 3:**
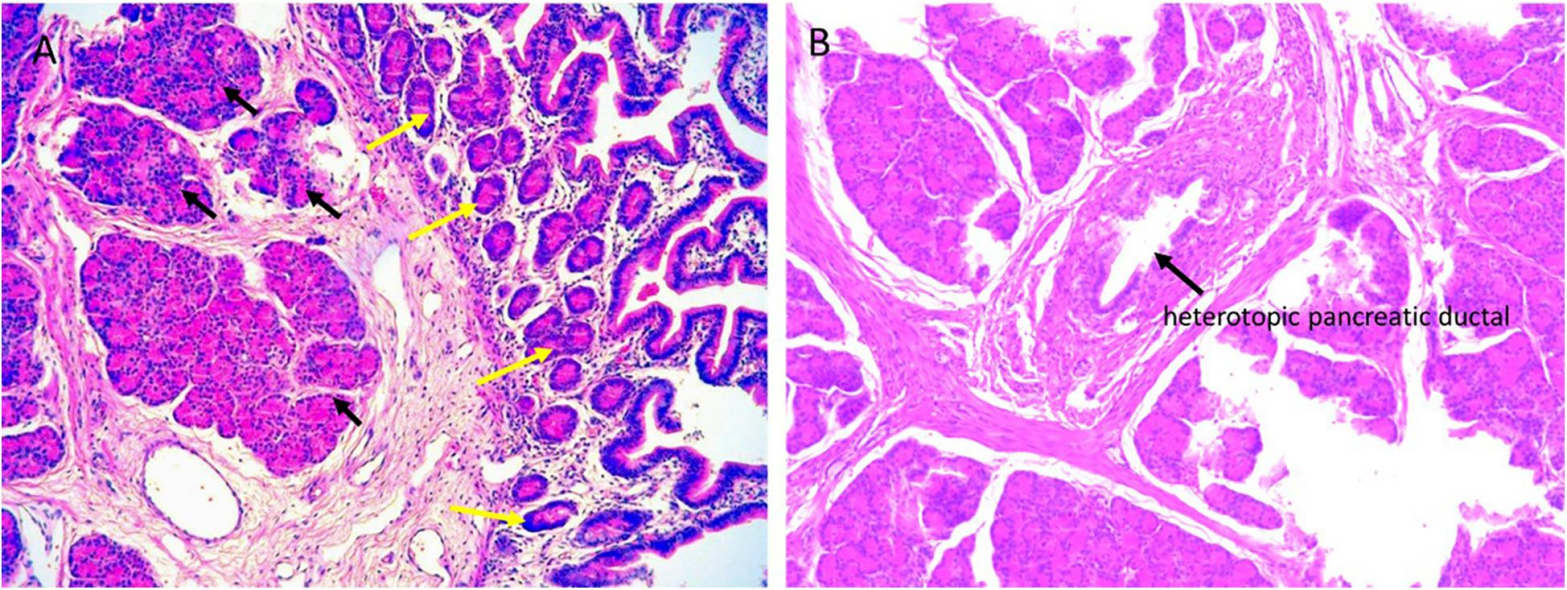
Histopathological analysis of the resected specimen. **(A)** Microscopically, the lesion consists of HP tissue (black arrow), including acini, islet cells, and HP tissue extending to the jejunal serosa (yellow arrow) (magnification × 200). **(B)** HP tissue and pancreatic ducts (black arrow) (magnification × 100). The histopathological features of the lesion are consistent with Heinrich Type I.

Based on the “double-bubble” sign and CDO diagnosis, emergency laparotomy was performed 72 h post-birth. Preoperative nasogastric decompression yielded 50 ml of bilious fluid, substantially reducing abdominal distension. Intraoperative exploration confirmed membranous duodenal atresia (Figure 2A), and a duodenoduodenostomy was performed. During the procedure, a systematic exploration of the entire gastrointestinal tract (from the stomach to the rectum) was conducted, and no other obstructive lesions were identified. Incidentally, a 10.0 × 6.0 mm mass adherent to the boundary between the jejunum and its mesentery was identified (Figure 2B). Given potential complication risks, the mass was surgically resected. To ensure complete excision of the lesion and prevent residual pancreatic tissue, approximately 2 cm of the adjacent normal jejunal wall was resected along with the mass. Subsequently, primary jejunojejunostomy was performed using an end-to-end anastomosis technique to restore intestinal continuity. Histopathological examination of the resected specimen confirmed the diagnosis of MHP. Hematoxylin-eosin (H&E) staining of the HP tissue revealed typical acinar, ductal, and islet structures, consistent with Heinrich Type I (Figure 3).

Postoperative laboratory investigations on day 3 revealed the following results: WBC 13.8 × 10⁹/L (45% neutrophils, 40% lymphocytes); RBC 4.9 × 10^12^/L; Hb 160.0 g/L; PLT 220.0× 10⁹/L; Na^+^ 136.0 mmol/L, K^+^ 3.9 mmol/L, Cl^−^ 96.0 mmol/L. Serum amylase was 65.0 U/L and lipase was 48.0 U/L. Hepatic and renal function panels were within normal limits. The postoperative recovery was uneventful. Prophylactic antibiotics were administered preoperatively and for the first three days after surgery. Minimal enteral feeding was initiated on postoperative day 3 and advanced to full feeding by day 7. The patient was discharged on postoperative day 8. Twelve-month follow-up ultrasound showed no evidence of recurrent HP. Written informed consent for the publication of this case report and any accompanying images was obtained from the neonate's parents prior to manuscript submission.

## 3 Discussion

HP refers to pancreatic parenchyma anatomically separate from the orthotopic pancreas, lacking continuity with the normal pancreas. First described by Jean Schultz in 1727, HP was not histologically confirmed until Klob's detailed characterization in 1859 (5). HP is typically discovered incidentally during autopsy, surgery, or endoscopy (1, 4). Current literature—primarily case reports and small series—lacks large clinicopathologic studies (6). This gap in the literature, combined with its frequent subclinical presentation, makes it difficult to determine the true incidence.

HP is more common in males than females, with peak onset typically occurring between ages 40 and 60 (3, 4). The most frequent sites for HP anomalies are the duodenum (30%), stomach (25%), and jejunum (15%), followed by Meckel's diverticulum (6%). Ileal involvement is rare (3%) (1). Dolan et al. (3) reported that among 212 patients with HP, 136 were male and 76 were female. The heterotopic tissue was located in the stomach (81 cases), duodenum (77 cases), or upper jejunum (33 cases). The remaining cases involved Meckel's diverticulum (6%), ileum (3%), and various other sites in small proportions. In contrast, Zhang et al. (4) observed a slight female predominance in a cohort of 184 patients with HP (male-to-female ratio: 0.94). This study found the stomach to be the most common location (97 cases, 52.7%), followed by the small intestine (48 cases, 26%), lesser omentum (18 cases, 10%), spleen/portal vein region (5 cases, 2.7%), mesentery (7 cases, 3.8%), distal esophagus (4 cases, 2.2%), hilar region (2 cases, 1%), and gallbladder (1 case, 0.5%). CDO-HP co-occurrence remains exceptionally rare, with pediatric MHP being particularly scarce—rarely reported in children <10 years and exceedingly rare in neonates (7).

Since most occurrences of HP are in the upper gastrointestinal tract, extraintestinal incidence is substantially lower. We searched PubMed from January 1980 to August 2025 using [(“heterotopic pancrea*” OR “ectopic pancrea*”) AND mesent*]. Reference lists of eligible articles were screened. We included English-language case reports/series describing mesenteric HP with clinical details; our case was also included. A total of 32 reports were identified (7–31) (Table 1). From the table, MHP demonstrated a striking female predominance (68.75% [22/32] female vs. 31.25% [10/32] male), contrasting sharply with the established male predominance in general HP. Anatomically, 78.125% (25/32) involved jejunal mesentery, while 12.5% (4/32) affected colonic mesentery. Three additional cases described MHP in small intestinal mesentery without precise localization (12, 15, 18). Pathologically, most were Heinrich type I, comprising acini, ducts, and islet cells.

**Table 1:** List of cases of mesenteric heterotopic pancreas in medical literature.

Source.	From	No.	Age	Sex	Location	Heinrich type	Methods of diagnosis	Clinical manifestation	Resection	Follow up
Koltai (7)	German	1	1.5 yr	F	Mesocolon	N/A	US + HP	Loss of appetite + right mid-abdominal mass	Yes	N/A
Fam (8)	America	1	12 yr	M	Jejunal mesentery	N/A	surgical exploration + HP	Abdominal pain around the umbilicus + nausea + vomiting	Yes	N/A
Iuchtman (9)	Israel	1	35 yr	F	Mesocolon	N/A	US + HP	Cystic lesions incidentally detected on ultrasound	Yes	N/A
Tornóczky (10)	Hungary	1	15 yr	F	Mesocolon	Tumor	CT + HP	Abdominal pain + bloating	Yes	N/A
Matsumoto (11)	Japan	1	3 yr	F	Mesocolon	N/A	US + CECT + HP	abdominal pain	Yes	20 months, asymptomatic
Silva (12)	America	1	57 yr	F	Mesentery	N/A	CECT + MRCP	Right back pain + full abdominal pressure + nausea	No	N/A
Shin (13)	Korea	1	38 yr	M	Jejunal mesentery	N/A	CECT + HP	One syncopal episode + black stools + no pressure in abdomen	Yes	9 months, no recurrence
Canbaz (14)	Turkish	1	75 yr	F	Jejunal mesentery	N/A	US + HP	Periumbilical pain, nausea, vomiting + elevated amylase and lipase	Yes	1year, no recurrence
Yang (15)	China	1	7 yr	F	Mesentery	Pancreatic blastoma	US + CT + MRI + HP	Abdominal pain + vomiting	Yes	3 years, no recurrence of tumor
Wong (16)	Canadian	1	67 yr	F	Jejunal mesentery	N/A	CECT + MRCP + HP	Epigastric tingling radiating to the back + Nausea + Vomiting + Elevated serum lipase	Yes	6 months, no recurrence
Wang (17)	China	1	3mo	F	Jejunal mesentery	N/A	Intraoperative Finding + HP	Asymptomatic + incidental intraoperative findings	N/A	N/A
Vyas (18)	India	1	19 yr	M	Mesentery	N/A	CECT + MRI	Abdominal pain + fever + easy fatigue	N/A	N/A
Ginsburg (19)	America	1	15 yr	F	Jejunal mesentery	I	CECT + HP	Total abdominal pain + elevated serum lipase and amylase	Yes	N/A
Seo (20)	Korea	7	Avg 50 yr (range 36–61)	3M/4F	Jejunal mesentery	N/A	CECT	Other diseases found on CT examination	No	26–77 months (median 39 months), asymptomatic
Borghol (21)	French	1	65 yr	M	Jejunal mesentery	N/A	CT	Asymptomatic + follow-up imaging for colon cancer	No	N/A
Navickas (22)	Lithuania	1	49 yr	M	Jejunal mesentery	I	CECT + HP	Asymptomatic + excludes prostate cancer metastasis incidental findings	Yes	N/A
Moreno (23)	Netherlands	1	58 yr	F	Jejunal mesentery	I	CECT + MRI + EUS - FNA + HP	Acute abdominal pain + nausea + vomiting	Yes	N/A
De (24)	Japan	1	55 yr	F	Jejunal mesentery	N/A	CT + MRCP + HP	Upper abdominal pain + elevated blood amylase and lipase	Yes	N/A
Tang (25)	Spanish	1	59 yr	F	Jejunal mesentery	N/A	MDCT + EUS- FNA + MRCP	Upper abdominal pain + elevated lipase + elevated CA19.9	No	1 month, Ca 19.9 and lipase normalized
Yamakawa (26)	China	1	12 yr	F	Jejunal mesentery	I	US + CECT	Abdominal pain + peritoneal irritation + intermittent vomiting	Yes	1 year, no recurrence
Aslan (27)	Turkish	1	44 yr	F	Jejunal mesentery	N/A	CECT	abdominal pain	No	2 years,no signs of malignancy
Xu (28)	African	1	7 yr	M	Jejunal mesentery	I	HP	Right lower abdominal pain + non-bilious vomiting	Yes	3 months, no recurrence
Sathiadoss (29)	Canadian	1	45 yr	F	Jejunal mesentery	II	CECT + HP	Nausea, non-bilious vomiting and mild epigastric pain (lasting 4 years)	Yes	N/A
Bullock (30)	America	1	43 yr	F	Jejunal mesentery	I	CECT + MRI + MRCP	Frequent abdominal pain + elevated lipase	Yes	N/A
Okamura (31)	Japan	1	61 yr	M	Jejunal mesentery	II	CECT + MRCP	Abdominal pain + 4 history of pancreatitis	Yes	2 years, no recurrence
Our case	China	1	3 d	F	Jejunal mesentery	I	Intraoperative Finding + HP	Asymptomatic + incidental intraoperative findings	Yes	1 year, asymptomatic

US, ultrasonography; HP, histopathology; F, female; M, male; No., number.

CT enhancement patterns reflect HP's microscopic composition: acinar-dominant lesions show homogeneous enhancement similar to or exceeding normal pancreas, while ductal-dominant lesions exhibit heterogeneous, reduced enhancement (32, 33). MHP demonstrates imaging characteristics analogous to orthotopic pancreas. Jejunal MHP typically appears as an elongated, lobulated lesion with a broad jejunal base (34). Mesenteric lesions show higher long-to-short diameter ratios (3.0) than gastric/proximal small bowel lesions (1.4–1.5) (20). MHP maximum diameters exceed gastric HP (4.4 cm vs. 1.8–2.7 cm), with longitudinal jejunal alignment more frequent than at other HP sites (20). Histopathologically, microscopic HP infiltrates jejunal walls into muscularis propria (13). In our case, HP extended to the serosa (Figure 3), suggesting MHP may exhibit extra-tubular growth.

In our review of the literature on MHP, CECT and MRCP proved most diagnostically valuable. Most patients exhibited normal orthotopic pancreas, while the HP typically manifested acute pancreatitis symptoms (12, 14, 23, 27, 30). Additionally, two cases of HP-related malignant transformation have also been reported (10, 15). Nevertheless, even in these presentations, differentiation from alternative conditions remains essential. Key differential diagnoses include acute appendicitis, perforated appendicitis, Meckel's diverticulitis (8, 28, 31), and para-duodenal hernia (19). When evaluating suspected gastrointestinal mesenchymal tumors, carcinoid tumors, lymphomas, and metastases—which may similarly present as homogeneous, well-enhanced soft tissue masses within the mesentery—CECT demonstrates limited reliability for distinguishing MHP (29). Although this patient's preoperative serum amylase and lipase levels were within normal limits and the mesenteric heterotopic pancreas (MHP) showed no secondary organic lesions, the future risk of severe complications—such as pancreatitis or malignant transformation—cannot be excluded. Given this potential for serious disease and malignant progression, local resection of the MHP represents the optimal clinical strategy for long-term prognosis (9, 13, 15, 29).

It is noteworthy that congenital duodenal atresia is frequently associated with other congenital anomalies, with congenital heart disease and Down syndrome being the most common (35). Other associated malformations include renal abnormalities and biliary atresia (36, 37). Therefore, conducting relevant baseline screening is crucial for patients with congenital duodenal atresia, both preoperatively and postoperatively (37). We also noted that Song et al. (38) previously reported a case of congenital duodenal atresia with coexisting heterotopic pancreas (HP), mirroring the dual pathological findings observed in our patient. This similarity raises a fundamental embryological question: Could aberrant migration or differentiation of the pancreatic bud disrupt duodenal recanalization during embryonic development? Importantly, this hypothesis does not amount to an inference of a causal relationship; it merely represents a potential embryological mechanism (38).

Bilious vomiting accompanied by delayed meconium passage in a neonate represents a clinical emergency, necessitating immediate referral to a specialized neonatal surgical center to prevent life-threatening complications such as intestinal ischemia, perforation, or septic shock. In the evaluation of a neonatal mesenteric mass or cyst, key differential diagnoses should include congenital enteric cyst, mesenteric lymphangioma, simple cyst, neoplastic cyst, and infectious cyst. In the present case, the mass was histopathologically confirmed to be HP without signs of metaplasia. An open surgical approach was adopted for this case, rather than a laparoscopic procedure. This decision was made after thorough team discussion, considering our limited experience with neonatal laparoscopic surgery. The primary goals were to ensure the safety of the anastomosis, minimize the risk of intraoperative complications, and optimize postoperative outcomes. The procedure was performed after obtaining informed consent from the child's guardians.

In conclusion, given the potential association between HP and duodenal atresia in asymptomatic cases, we propose systematic screening for HP in all neonates with this condition. If identified, concurrent prophylactic resection of the HP lesion during the repair of duodenal atresia is recommended to mitigate the risk of long-term complications, including pancreatitis and gastrointestinal bleeding.

## Data Availability

The original contributions presented in the study are included in the article/Supplementary Material, further inquiries can be directed to the corresponding author.
